# Kinetic modeling of the plasma pharmacokinetic profiles of ADAMTS13 fragment and its Fc-fusion counterpart in mice

**DOI:** 10.3389/fphar.2024.1352842

**Published:** 2024-03-25

**Authors:** Heechun Kwak, Min-Soo Kim, Suyong Kim, Jiyoung Kim, Yasunori Aoki, Suk-Jae Chung, Hyun-Ja Nam, Wooin Lee

**Affiliations:** ^1^ College of Pharmacy and Research Institute of Pharmaceutical Sciences, Seoul National University, Seoul, Republic of Korea; ^2^ Discovery Unit, Research and Early Development Department, GC Biopharma Corp, Yongin-si, Republic of Korea; ^3^ Laboratory of Quantitative System Pharmacokinetics/Pharmacodynamics, Josai International University, Tokyo, Japan; ^4^ Drug Metabolism and Pharmacokinetics, Research and Early Development, Cardiovascular, Renal and Metabolism (CVRM), BioPharmaceuticals R&D, AstraZeneca, Gothenburg, Sweden

**Keywords:** ADAMTS13, kinetic modeling, pharmacokinetics, Fc-fusion, recombinant proteins

## Abstract

**Introduction:** Fusion of the fragment crystallizable (Fc) to protein therapeutics is commonly used to extend the circulation time by enhancing neonatal Fc-receptor (FcRn)-mediated endosomal recycling and slowing renal clearance. This study applied kinetic modeling to gain insights into the cellular processing contributing to the observed pharmacokinetic (PK) differences between the novel recombinant ADAMTS13 fragment (MDTCS) and its Fc-fusion protein (MDTCS-Fc).

**Methods:** For MDTCS and MDTCS-Fc, their plasma PK profiles were obtained at two dose levels following intravenous administration of the respective proteins to mice. The plasma PK profiles of MDTCS were fitted to a kinetic model with three unknown protein-dependent parameters representing the fraction recycled (FR) and the rate constants for endocytosis (k_up_, for the uptake into the endosomes) and for the transfer from the plasma to the interstitial fluid (k_pi_). For MDTCS-Fc, the model was modified to include an additional parameter for binding to FcRn. Parameter optimization was done using the Cluster Gauss-Newton Method (CGNM), an algorithm that identifies multiple sets of approximate solutions (“accepted” parameter sets) to nonlinear least-squares problems.

**Results:** As expected, the kinetic modeling results yielded the FR of MDTCS-Fc to be 2.8-fold greater than that of MDTCS (0.8497 and 0.3061, respectively). In addition, MDTCS-Fc was predicted to undergo endocytosis (the uptake into the endosomes) at a slower rate than MDTCS. Sensitivity analyses identified the association rate constant (k_on_) between MDTCS-Fc and FcRn as a potentially important factor influencing the plasma half-life *in vivo*.

**Discussion:** Our analyses suggested that Fc fusion to MDTCS leads to changes in not only the FR but also the uptake into the endosomes, impacting the systemic plasma PK profiles. These findings may be used to develop recombinant protein therapeutics with extended circulation time.

## Introduction

ADAMTS13 (the 13th member of a disintegrin-like and metalloprotease with thrombospondin type 1 motif) is a plasma protease (about 190 kDa) that cleaves the Tyr1605–Met1606 bond in the A2 domain of von Willebrand factor (vWF) (Lancellotti and De Cristofaro, 2011; Xiang et al., 2011). Genetic deficiency or immune-mediated neutralization of ADAMTS13 activity can cause congenital or immune-mediated thrombotic thrombocytopenic purpura (cTTP or iTTP), a hematological disorder frequently associated with multiple organ failure (Sadler, 2017). Currently, human ADAMTS13 and its recombinant versions are under development as potential TTP therapy (Tersteeg et al., 2015; Scully et al., 2017).

**FIGURE 1:**
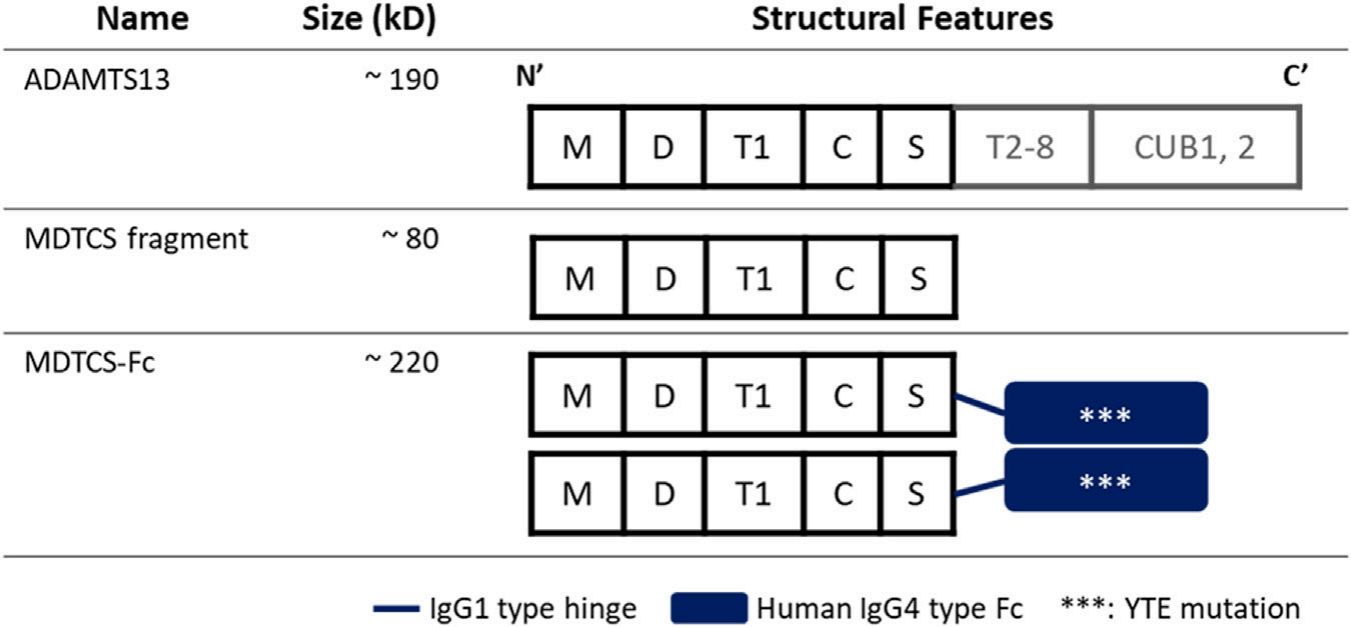
Diagrams depicting the structures of ADAMTS13, MDTCS, and MDTCS-Fc. The MDTCS fragment is a truncated form of ADMTS13 and consists of the M, D, T1, C, and S domains essential to cleave vWF multimers. MDTCS-Fc consists of MDTCS, an IgG1 type hinge, and an IgG4 type Fc harboring YTE mutations to improve the FcRn binding affinity. M: metalloprotease domain, D: disintegrin-like domain, T1: the first thrombospondin type 1 repeat, C: Cys-rich domain, S: spacer domain, T2-8: the second to eighth TSP1 repeats, CUB: Complement c1r/c1s, sea Urchin epidermal growth factor, and bone morphogenetic protein, Fc: Fragment crystallizable.

The structure-function understanding of ADAMTS13 has been essential in designing various recombinant versions. As depicted in Figure 1, ADAMTS13 has a conserved MDTCS domain organization at the N-terminus (Lancellotti and De Cristofaro, 2011). The fragment containing the MDTCS domain only (about 80 kDa) retains the metalloprotease activity to cleave vWF multimers (Ai et al., 2005) and may offer therapeutic benefits even for iTTP patients by escaping the capture by the autoantibodies which recognize the C-terminus of ADAMTS13 (Tersteeg et al., 2016). To enhance the druggability and prolong the circulation time, recombinant proteins were designed by fusing the MDTCS fragment with the fragment crystallizable (Fc) region of immunoglobulin G (IgG) 4. Through activity-based screening of varying recombinant proteins, MDTCS-Fc (about 220 kDa) was selected for further evaluation. As depicted in Figure 1, MDTCS-Fc is a dimeric protein with the MDTCS fragment, the IgG1 type hinge, and the fused Fc domain harboring the mutations in three amino acids [M252Y/S254T/T256E (YTE)]. The YTE mutations are reported to increase the binding to the mouse neonatal Fc-receptor (FcRn) by approximately 10-fold and to prolong the serum half-life in the cynomolgus monkey by approximately 4-fold, compared to its wild-type form (Dall'Acqua et al., 2002; Dall'Acqua et al., 2006).

Mechanistic kinetic modeling has proven valuable in drug development, especially in bridging the gap between *in vitro* and *in vivo* data. For various Fc-fusion protein therapeutics, mechanistic kinetic models have been developed to incorporate the FcRn recycling pathway and applied to analyze the systemic pharmacokinetic (PK) profiles (Yuan et al., 2018; Hardiansyah and Ng, 2022). These mechanistic kinetic models have been increasingly applied in guiding the design of Fc-engineered protein therapeutics, selecting optimal doses, and aiding in inter-species scale-up. In most cases, the direct comparison between the Fc-fusion and native forms was lacking.

This study obtained the plasma PK profiles of MDTCS and MDTCS-Fc after intravenous (i.v.) administration at the two dose levels in mice. For the obtained PK data, the kinetic modeling was performed using the Cluster Gauss-Newton method (CGNM), an algorithm that identifies multiple sets of approximate solutions (“accepted” parameter sets) to nonlinear least-squares problems (Aoki and Sugiyama, 2023). The current results may offer insights into the factors impacting the plasma PK profiles of Fc-fusion proteins and considerations in developing Fc-fusion therapeutic drugs.

## Materials and Methods

### Materials

MDTCS and MDTCS-Fc were produced in Chinese hamster ovary (CHO) cells by a serum-free cell culture method (GC Biopharma, Republic of Korea) and their respective activity was measured with TECHNOZYM^®^ ADAMTS13 activity enzyme-linked immunosorbent assay (ELISA) kit (Technoclone Herstellung von Diagnostika und Arzneimitteln GmbH, Vienna, Austria) following the recommended protocol. A human ADAMTS13 DuoSet ELISA kit (R&D systems, Minneapolis, United States) was used to measure the concentrations of MDTCS or MDTCS-Fc in the plasma samples collected from the mice receiving MDTCS or MDTCS-Fc. The 96-well plates [Enzyme Immunoassay/Radioimmunoassay (EIA/RIA) plate or Greiner 96 well plate] were from Merck KGaA (Darmstadt, Germany) and 1-Step™ Ultra 3,3′,5,5′-Tetramethylbenzidine (TMB)-ELISA Substrate Solution was from Thermo Scientific (Massachusetts, United States). The biotinylated mouse FcRn protein was from Acro Biosystems (Delaware, United States). The streptavidin biosensor was from Sartorius AG (Göttingen, Germany).

### Quantitation of MDTCS or MDTCS-Fc by the ELISA

Each well of 96-well EIA/RIA plates was coated with the capture antibody (Ab) and incubated overnight at 2°C—8°C, followed by rinsing with the washing buffer [phosphate-buffered saline (PBS) containing 0.1% tween20] using a microplate washer. The rinsing step was repeated between each step. The plates were incubated with the blocking buffer [PBS containing 1% bovine serum albumin (BSA)] for 2 h at room temperature (r.t.) (unless mentioned otherwise). The standards containing the known concentrations of MDTCS or MDTCS-Fc were prepared by serial dilution using the blocking buffer and combined with the mouse plasma. The plasma samples from mice that received i.v. dosing of MDTCS or MDTCS-Fc were diluted with blocking buffer to bring them within the standard concentration range before loading them into each well. The plates were incubated for 2 h with 400 rpm shaking, followed by the incubation with the detection Ab (for 2 h with 400 rpm shaking) and with streptavidin-HRP (for 20 min). The substrate solution was added to washed plates and incubated for 20 min. After stopping the reaction by adding 1N H_2_SO_4_, the absorbance was measured at 450 nm and 570 nm (to subtract the background signal) using a SpectraMax iD5 microplate reader (Molecular Devices, California, United States). For quantitation, the standard curves were fitted to a four-parameter logistic equation (SoftMax Pro version 7.1, Molecular Devices). The final concentration was calculated using the dilution factor of each sample that was used.

### Assessment of the binding affinity of MDTCS-Fc to mouse FcRn

The binding affinity of MDTCS-Fc to mouse FcRn was measured using the biolayer interferometry (BLI) system, as reported previously (Neuber et al., 2014). The biotinylated mouse FcRn protein [1.0 μg/mL, diluted with the assay buffer (25 mM acetate, 25 mM NaH_2_PO_4_, 150 mM NaCl, 0.01% Tween-20, pH 6.0)] was immobilized on the streptavidin biosensor tip. The immobilization of the mouse FcRn was verified by the spectral shift of 0.8 ± 0.2 nm arising from the changes in thickness. The solutions of MDTCS-Fc (the concentrations ranging from 0.625 to 20 nM, diluted with the assay buffer) were transferred to Greiner 96 well plates, which were then placed on the Octet Qke (Sartorius AG). At the association phase (to assess the association rate constant k_on_), MDTCS-Fc solutions were allowed to bind to mouse FcRn-immobilized biosensors for 5 min. At the dissociation phase (to assess the dissociation rate constant k_off_), the mouse FcRn-immobilized biosensors complexed with MDTCS-Fc were incubated in the assay buffer for 5 min. To subtract the non-specific binding signal between MDTCS-Fc and the streptavidin biosensor, the procedure was conducted as described above, but using the biosensors lacking the immobilized mouse FcRn. The sensorgrams were analyzed with the data analysis program version 11.1 (Sartorius AG).

### The plasma PK study of MDTCS or MDTCS-Fc in mice following i.v. dosing

The animal study protocol was approved by the Institutional Animal Care and Use Committee of KPC (KPC-E2021124, Gyeonggi-do, Republic of Korea). The two different doses (160 or 320 IU/kg) of MDTCS or MDTCS-Fc were intravenously given to C57BL/6 mice [7-week-old, of either gender; Orient Bio (Gyeonggi-do, Republic of Korea)]. As the specific activity differed between MDTCS and MDTCS-Fc (410.4 and 4054.4 IU/nmol, respectively), the converted molar doses were as follows: for MDTCS, 0.39 and 0.78 nmol/kg, and for MDTCS-Fc, 0.0394 and 0.0788 nmol/kg. After i. v. dosing, blood samples were collected by cardiac puncture (n = three to four per sampling time) at 0.083, 0.25, 0.5, 1, 2, 4, 8, 12, 24, 48, 72, 96, or 168 h. The blood samples were transferred to a 1.5 mL tube containing 10% sodium citrate, followed by centrifugation at 2,000 g for 20 min at 4 °C. The resulting plasma samples were stored at −75 (±5)°C until the quantitation of MDTCS or MDTCS-Fc using the ELISA described in the previous section. In the case of the samples with the signals below the lowest concentrations of the calibration standards, those samples were regarded as missing values (Beal, 2001). At each sampling time, the average concentrations of MDTCS or MDTCS-Fc were used to calculate the relevant PK parameters by non-compartmental analysis (naive pooled data approach) (WinNonlin software version 8.4, Certara, New Jersey, United States).

### Development of the mechanistic kinetic models for MDTCS and MDTCS-Fc

**FIGURE 2:**
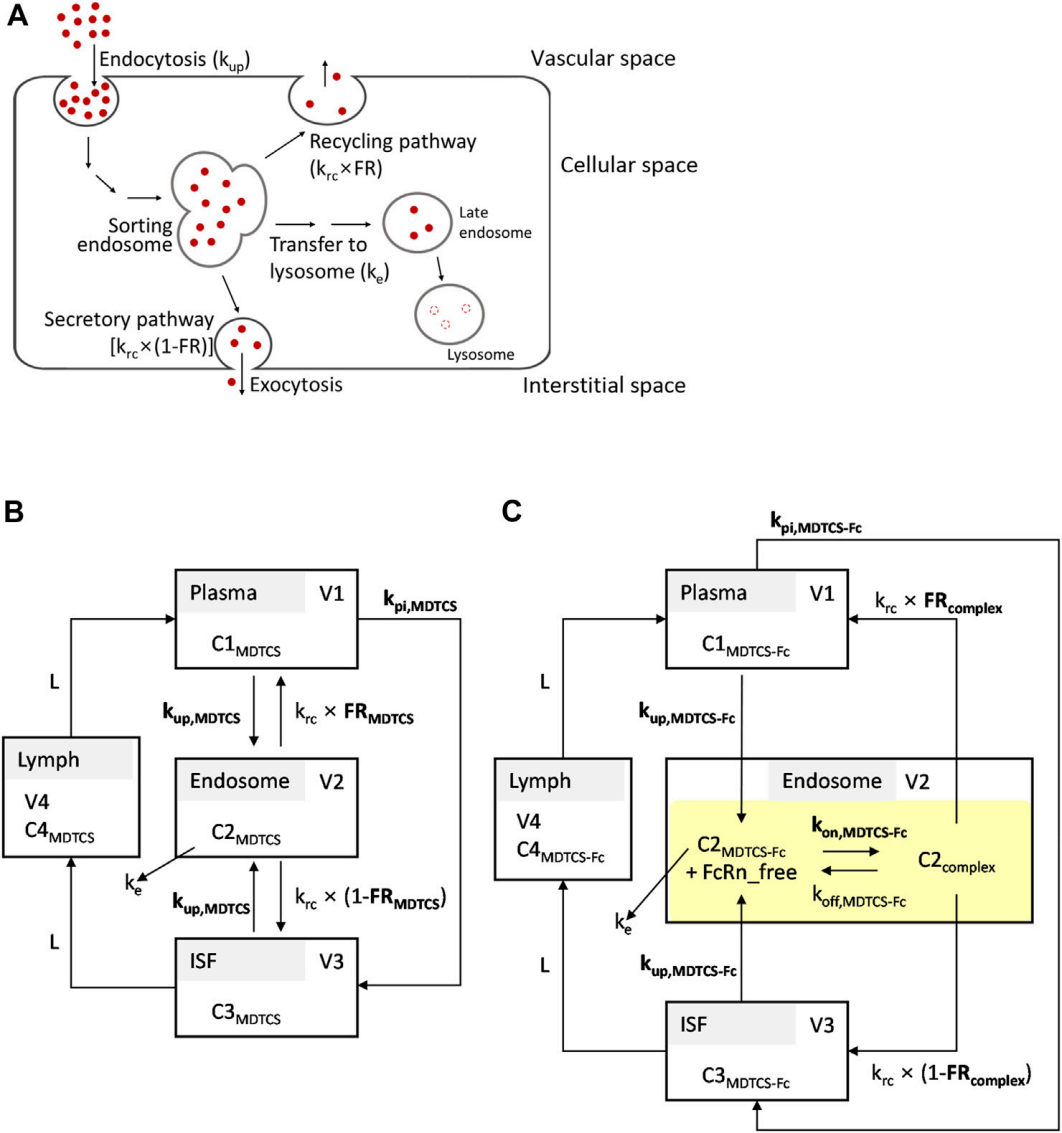
**(A)** Simplified scheme depicting the processing of therapeutic proteins in the endosomes and lysosomes. The transport of therapeutic proteins may occur in either direction: from vascular to interstitial space or *vice versa*. **(B,C)** Structures of the kinetic models for MDTCS and MDTCS-Fc used in the current study. The kinetic models incorporated the following processes: i) endocytosis (k_up_), ii) recycling (k_rc_), iii) secretion (k_rc_, the opposite side direction), and iv) the transfer from the endosome to the lysosome (k_e_). The k_pi_ is the rate constant of the transfer from the plasma to ISF. For MDTCS-Fc, the model incorporated the FcRn binding in the endosome (highlighted in yellow). Protein-dependent parameters are denoted in bold. Refer to the text and Table 1 for abbreviations.

To obtain kinetic insights into the *in vivo* handling of MDTCS and MDTCS-Fc, the mechanistic kinetic model was developed by considering the following processes. Upon cellular entry via endocytosis, MDTCS or MDTCS-Fc is located to acidic endosomes. At the sorting endosome, these proteins typically face the following pathways: 1) the lysosomal degradation, 2) the recycling, and 3) the secretion to the opposite side (transcytosis) (Figure 2A) (Serra and Sundaram, 2021). By modifying the model structures reported previously (Hardiansyah and Ng, 2022), the kinetic models for MDTCS and MDTCS-Fc were constructed to include the four compartments for plasma, endosome, interstitial fluid (ISF), and lymphatic fluid, as depicted in Figures 2B, C, respectively.

The following assumptions were made: Firstly, the process by which proteins enter the endosome via endocytosis takes place at the same protein-dependent rate constants regardless of the direction (i.e., the uptake of MDTCS or MDTCS-Fc into the endosome from either plasma or ISF can be described by k_up,MDTCS_ and k_up,MDTCS-Fc_, respectively); secondly, the return of proteins from the endosome to plasma takes place at the protein-independent rate constant of k_rc_ multiplied by the protein-dependent recycling fractions of FR_MDTCS_ and FR_complex_; thirdly, the return of MDTCS or MDTCS-Fc from the endosome to ISF takes place at the protein-independent rate constant of k_rc_ multiplied by (1—FR_MDTCS_) and (1—FR_complex_), respectively. Lastly, the transfer of MDTCS or MDTCS-Fc from the plasma to ISF takes place at the protein-dependent rate constant of k_pi,MDTCS_ or k_pi,MDTCS-Fc_, respectively (Hardiansyah and Ng, 2022). The transfer from ISF to lymph and that from lymph to plasma takes place via the lymph flow rate (L).

For MDTCS-Fc, its binding to FcRn and recycling was described using the equilibrium dissociation constant [K_D_, defined as k_off_ (the dissociation rate constant) divided by k_on_ (the association rate constant)]. The unbound MDTCS-Fc and MDTCS in the endosome were assumed to transfer to the lysosome (processing to the late endosome) at a rate described by k_e_ (assumed same for both MDTCS and MDTCS-Fc), fixed based on the reported value following the analysis by Yuan et al. (2018).

Table 1 lists the fixed system-dependent, protein-independent, and protein-dependent parameters optimized during the kinetic modeling (three and four parameters for MDTCS and MDTCS-Fc, respectively). The parameter describing the process entering the endosomes (k_up_) was assumed to be protein-dependent, considering that the processes are likely impacted by the protein characteristics such as the net charge, molecular weight, and post-translational modification (Duncan et al., 1981; Boswell et al., 2010; Hardiansyah and Ng, 2022). The constructed mechanistic kinetic models of MDTCS and MDTCS-Fc included four and six ordinary differential equations (ODEs), respectively (provided in the Supplementary Method).

**TABLE 1:** List of the fixed and protein-dependent parameters optimized during the mechanistic kinetic modeling of MDTCS and MDTCS-Fc.

Parameter	Description	Unit	Initial range (min, max)	Notes and references for the base values
Fixed parameter				
V1	plasma volume	mL	0.85	Zhao et al. (2015)
V2	endosomal volume	mL	0.1	Garg and Balthasar, (2007)
V3	interstitial fluid volume	mL	4.35	Zhao et al. (2015)
V4	lymph volume	mL	1.6	Zhao et al. (2015)
L	lymph flow	mL×hr^-1^	0.12	Zhao et al. (2015)
k_rc_	the endosomal recycling rate constant	hr^-1^	5.1975	Yuan et al. (2018)
k_e_	the transfer rate constant to the lysosome	hr^-1^	0.5396	Yuan et al. (2018)
K_D_	equilibrium dissociation constant between MDTCS-Fc and mouse FcRn at pH 6.0	nM	0.144	Experimentally measured
FcRn_total	total concentration of FcRn	μM	40	The effective concentration of free FcRn binding sites in mice was predicted based on the IgG concentration saturating the FcRn binding sites (Ferl et al., 2005)
For MDTCS				
k_pi,MDTCS_	The transfer rate constant from plasma to interstitial fluid	hr^-1^	(0.001, 100)	-
k_up,MDTCS_	Pinocytosis rate constant	hr^-1^	(0.0005, 5.0)	The estimated uptake rate of the IgG into the endosome via the fluid phase (Garg and Balthasar, 2007; Yuan et al., 2018)
FR_MDTCS_	Fraction of recycled therapeutics to plasma	NA	(0.0001, 0.9999)	A possible range for a fraction was used
For MDTCS-Fc				
k_pi,MDTCS-Fc_	The transfer rate constant from plasma to interstitial fluid	hr^-1^	(0.001, 100)	-
k_up,MDTCS-Fc_	Pinocytosis rate constant	hr^-1^	(0.0005, 5.0)	The estimated uptake rate of the IgG into the endosome via the fluid phase (Garg and Balthasar, 2007; Yuan et al., 2018)
k_on,MDTCS-Fc_	The association rate constant of FcRn binding in acidic endosomes (measured at pH 6.0)	nM^-1^×hr^-1^	(0.00048, 4.87)	The base value of 0.0487 was experimentally measured by the BLI system in the current study
FR_complex_	Fraction of recycled therapeutics to plasma	NA	(0.0001, 0.9999)	A possible range for a fraction was used

### Parameter optimization by the cluster gauss-newton method (CGNM)

CGNM can yield multiple possible solutions to nonlinear least-squares problems (Aoki et al., 2022; Aoki and Sugiyama, 2023) and was used by the previous physiologically-based PK (PBPK) modeling (Koyama et al., 2021; Mochizuki et al., 2022; Yoshikado et al., 2022; Lee et al., 2023) and pharmacodynamic modeling (Niu et al., 2021). The CGNM finds multiple sets of parameters by repeating the parameter estimations from multiple initial iterates randomly selected between the lower and upper ranges based on the reported or user-defined values. For parameters optimization, the initial range was set as the base value multiplied by 10^−2^ to 10^2^, except for the fraction recycled (FR) whose range was set as 0.0001 and 0.9999 (Table 1). Then, with the initial iterates, the CGNM finds the best-fit parameters with the minimum sum of squares residual (SSR), as shown below.

$$SSR=\sum_{i=1}^{n}{\left(\log_{10}y_{obs,i}-\log_{10}y_{model-predicted,i}\right)}^{2}$$

(*n*, the number of observations; *y*_*obs,i*_, the *i*th observed value; *y*_*model-predicted,i*_, the *i*th model-predicted value).

The rxode2 package (version 2.0.11) was used to integrate a set of ODEs of mechanistic kinetic models, and CGNM was performed in RStudio (version 2022.07.2 + 576) with CGNM package (version 0.5.1). We set the number of initial parameter combinations (num_minimizersToFind) to 1,000 and the number of iteration (num_iteration) to 100 according to the user manual, while the rest of the running conditions was kept as default conditions. Then the elbow method was used to find the “acceptable” maximum SSR. To obtain accepted approximate minimizers, Grubbs’ test for outliers was used (alpha = 0.05).

## Results

### *In vitro* binding kinetics between MDTCS-Fc and mouse FcRn

**FIGURE 3:**
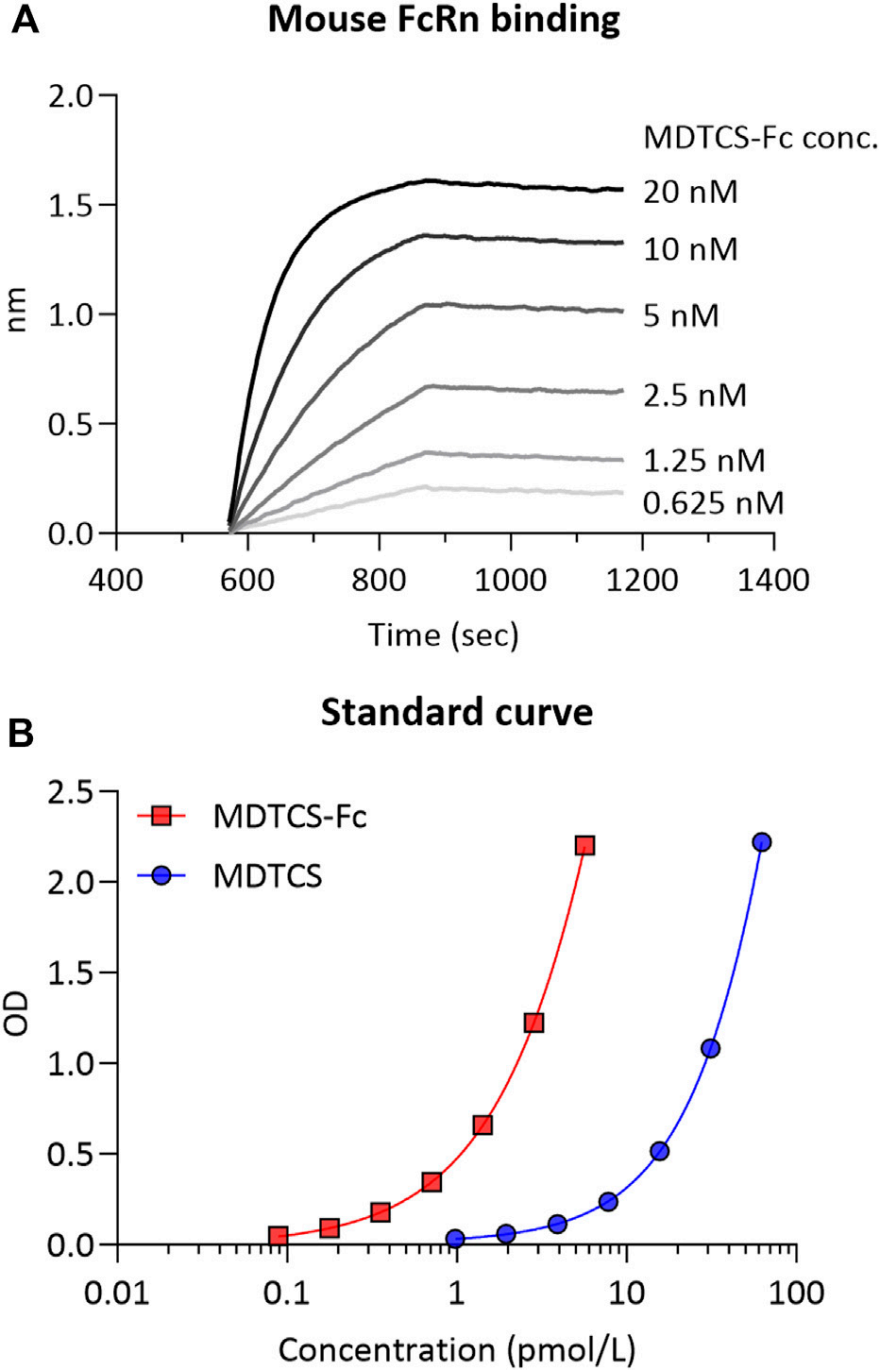
**(A)** The representative binding sensorgram of MDTCS-Fc to mouse FcRn. The binding sensorgram was recorded at pH 6.0 with the BLI system. **(B)** The representative standard curve was obtained from the ELISA for MDTCS-Fc and MDTCS. The standard concentration ranges were 0.0888–5.68 pM for MDTCS-Fc and 0.977—62.5 pM for MDTCS. Factoring in the dilution factor, the lower limit of quantitation (LLOQ) of MDTCS-Fc and MDTCS was calculated to be 0.888 pM and 9.77 pM, respectively.

To capture the binding of MDTCS-Fc and mouse FcRn in acidic endosomes, the *in vitro* binding kinetics were assessed between mouse FcRn and MDTCS-Fc at pH 6.0 (Figure 3A). The analyses of BLI sensograms yielded the following parameters: k_off_ and k_on_ values of 0.00704 ± 0.00148 h^-1^ and 0.0487 ± 0.0027 nM^-1^×h^-1^, respectively [yielding the equilibrium dissociation constant (K_D_, k_off_/k_on_) of 0.144 ± 0.022 nM]. This K_D_ value of 0.144 nM is not far from the anticipated value based on the literature. For the human IgG4 (with no YTE mutations), the reported K_D_ value for binding to mouse FcRn was about 6 nM when measured using the BLI method (Neuber et al., 2014) and the YTE mutations were reported to enhance the binding affinity by ∼ 10-fold (Dall'Acqua et al., 2006).

### Plasma PK profiles of MDTCS or MDTCS-Fc in mice

The concentrations of MDTCS or MDTCS-Fc in mouse plasma samples were quantified with the previously developed ELISA method: The working concentration ranges for MDTCS and MDTCS-Fc were 0.977–62.5 pM and 0.0888—5.68 pM, respectively, based on the criteria for accuracy and precision (Azadeh et al., 2017) (Figure 3B, Supplementary Table S1).

**FIGURE 4:**
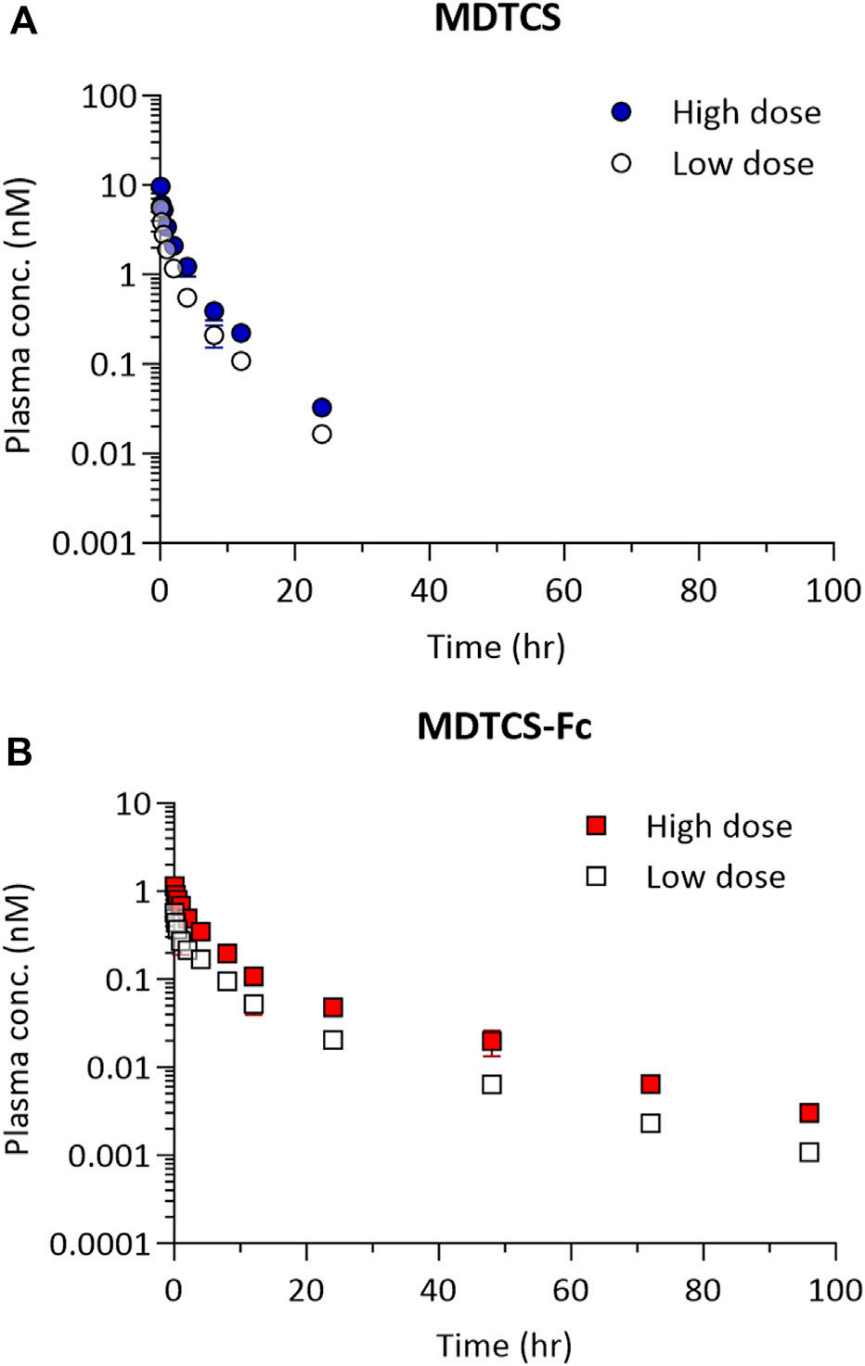
The plasma pharmacokinetic profiles of MDTCS **(A)** after a single i.v. dosing of 0.39 or 0.78 nmol/kg and MDTCS-Fc **(B)** after a single i.v. dosing of 0.0394 or 0.0788 nmol/kg in mice. Each data point represents the mean concentration with the corresponding standard deviation, measured using the plasma samples collected via heart puncture (n = three to four mice per time point).

At the two dose levels tested (0.39 and 0.78 nmol/kg), the plasma concentrations of MDTCS in mice declined rapidly, not quantifiable after 24 h post-dosing (Figure 4A). In contrast, at the two dose levels tested (0.0394 and 0.0788 nmol/kg), the plasma concentrations of MDTCS-Fc were quantifiable up to 96 h post-dosing (Figure 4B). For both MDTCS and MDTCS-Fc, the dose-normalized PK profiles were nearly overlapping (Supplementary Figure S1). The PK parameters obtained from non-compartmental analysis also indicated dose-proportional increases in the systemic exposure of MDTCS and MDTCS-Fc. The half-lives (t_1/2,terminal_) of MDTCS-Fc were 3.9-fold longer than those of MDTCS, and the total clearance (CL) of MDTCS-Fc was 3.1-fold slower than that of MDTCS (Table 2).

**TABLE 2:** Pharmacokinetic parameters of MDTCS and MDTCS-Fc following intravenous dosing at the two dose levels in mice. The PK parameters were calculated based on the plasma concentration-time profiles by non-compartmental analysis.

Parameter	Units	Dose (nmol/kg)			
		MDTCS		MDTCS-Fc	
		0.39	0.78	0.0394	0.0788
t_1/2, terminal_	hr	4.367	4.434	16.918	17.680
AUC_last_	hr×nM	9.508 (0.25)	17.889 (0.49)	2.745 (0.08)	6.148 (0.11)
AUC_inf_	hr×nM	9.613	18.098	2.771	6.225
CL	L×kg^-1^×hr^-1^	0.0406	0.0431	0.01422	0.01266
MRT	hr	3.654	3.867	12.87	14.54
V_ss_	L×kg^-1^	0.1483	0.1667	0.1829	0.1840

Numbers in the parentheses indicate standard errors associated with estimated parameters computed using the sparse data option (WinNonlin, version 8.4, certara, NJ, United States).

### Mechanistic kinetic modeling applied to the plasma PK profiles of MDTCS and MDTCS-Fc

**FIGURE 5:**
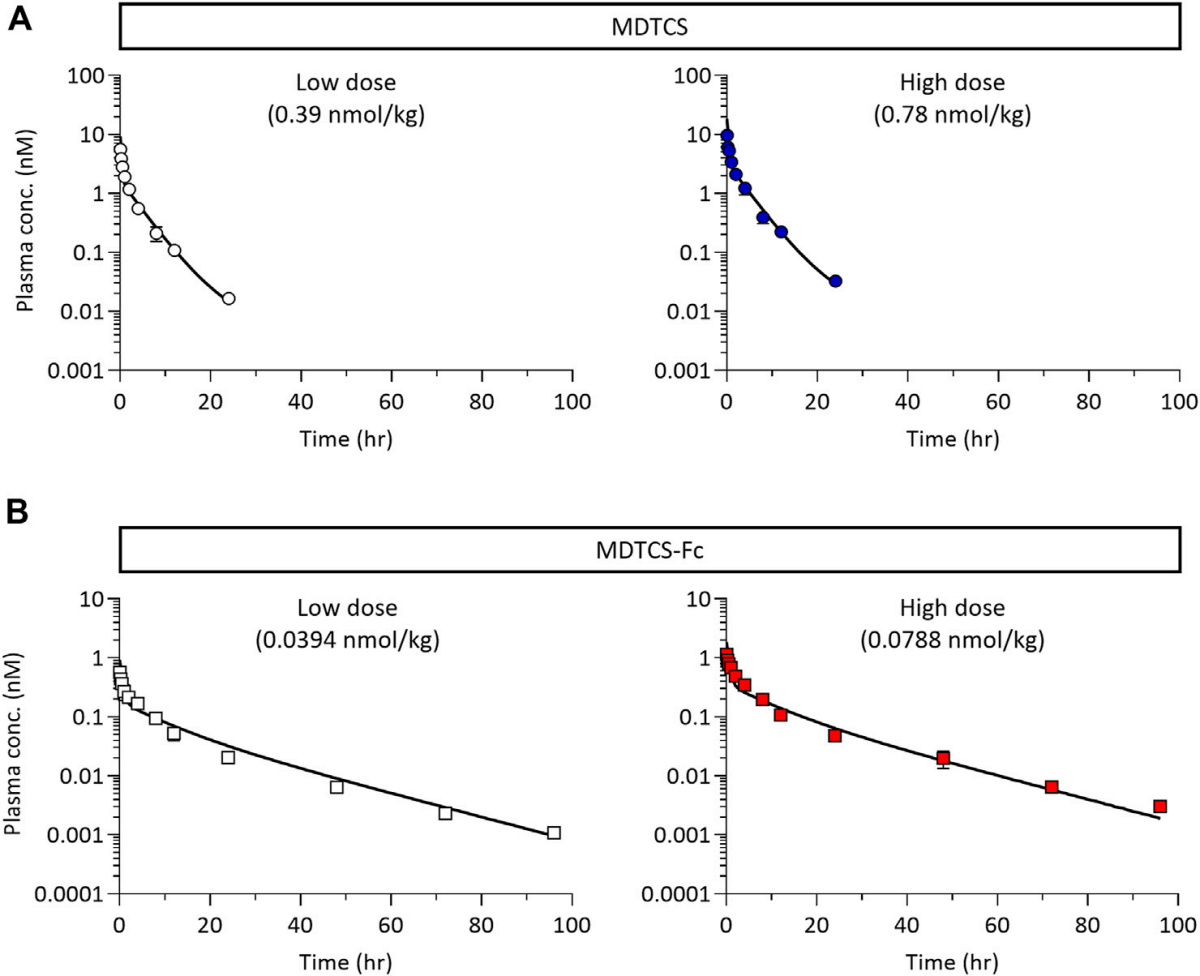
**(A)** The plasma pharmacokinetic profiles of MDTCS (after a single i. v. dosing of 0.39 and 0.78 nmol/kg) with the optimized parameter sets (n = 182) via CGNM analysis. The 182 profiles were nearly overlapping and appeared as a single line. The symbols represent the observed data of the average plasma concentrations of MDTCS, as shown Figure 4A. **(B)** The plasma pharmacokinetic profiles of MDTCS-Fc (after a single i. v. dosing of 0.0394 and 0.0788 nmol/kg) with the optimized parameter sets (n = 215) via CGNM analysis. The 215 profiles were nearly overlapping and appeared as a single line. The symbols represent the observed data of the average plasma concentrations of MDTCS-Fc, as shown in Figure 4B.

Using the kinetic model depicted in Figure 2B, the CGNM-based parameter optimization was performed to capture the plasma PK profiles of MDTCS. The results yielded 182 sets of the “accepted” parameters (below the cut-off SSR value of 0.10687), all of which well captured the observed PK profiles of MDTCS at both dose levels (0.39 and 0.78 nmol/kg) (Figure 5A): the simulated PK profiles using 182 sets of the accepted parameters were nearly overlapping and appeared as an almost single line. The two optimized parameters (FR_MDTCS_ and k_up,MDTCS_) were distributed in a narrow range, and the k_pi,MDTCS_ parameter was close to zero (Table 3, Supplementary Figure S2A). When simulation was performed using the parameter set of rank 1 (with the lowest SSR value), the model-predicted PK profiles yielded slightly greater AUC_0–24h_ values by 17.5% [9.51 (observed) vs.11.17 (model-predicted) nM×h] and 24.9% [17.89 (observed) vs. 22.34 (model-predicted) nM×h] for the dose levels of 0.39 and 0.78 nmol/kg, respectively.

**TABLE 3:** Summary of the accepted parameter values by the CGNM run analyzing the plasma PK data from two doses of MDTCS and MDTCS-Fc.

Proteins administered	Parameter	Value			
		Rank 1	Min	Max	Median
MDTCS	FR_MDTCS_	0.3061	0.3059	0.3062	0.3060
	k_up,MDTCS_ (hr^-1^)	4.012	4.011	4.013	4.012
	k_pi,MDTCS_ (hr^-1^)	2.126 × 10^−9^	1.691 × 10^−286^	3.254 × 10^−5^	1.911 × 10^−10^
MDTCS-Fc	FR_complex_	0.8497	0.8373	0.8661	0.8503
	k_up,MDTCS-Fc_ (hr^-1^)	0.5296	0.5219	0.5375	0.5296
	k_pi,MDTCS-Fc_ (hr^-1^)	1.191	1.184	1.201	1.191
	k_on,MDTCS-Fc_ (nM^-1^×hr^-1^)	6.729 × 10^−5^	6.617 × 10^−5^	6.838 × 10^−5^	6.729 × 10^−5^
	k_off,MDTCS-Fc_ (hr^-1^)^a^	9.690 × 10^−6^	9.529 × 10^−6^	9.846 × 10^−6^	9.690 × 10^−6^

^a^ k_off,MDTCS-Fc_ was not optimized but calculated as a secondary parameter using the relationship of K_D_ × k_on,MDTCS-Fc_.

For MDTCS-Fc, the kinetic model included an additional unknown parameter, as depicted in Figure 2C. The results yielded 215 sets of the “accepted” parameters (below the cut-off SSR value of 0.32674), all of which well captured the observed PK profiles of MDTCS-Fc at both dose levels (0.0394 and 0.0788 nmol/kg) and yielded an apparent single line (Figure 5B). The four optimized parameters (FR_complex_, k_up,MDTCS-Fc_, k_pi,MDTCS-Fc_, and k_on,MDTCS-Fc_) were distributed in a tight range (Table 3, Supplementary Figure S2B). The AUC_0–96h_ values were comparable between the observed data and the simulated PK profiles using the rank 1 parameters: the differences were 11.1% [2.745 (observed) vs. 3.049 (model-predicted) nM×h] and 0.8% [6.148 (observed) vs. 6.096 (model-predicted) nM×h], respectively.

When the rank 1 parameter values (with the smallest SSR) were compared, FR_complex_ (0.8497) was 2.8 times to FR_MDTCS_ (0.3061), as expected for Fc-fused proteins (Table 3). The rate constant k_up_ describing the uptake of the protein from either the plasma or ISF to endosomes was predicted to be 7.58 times smaller for MDTCS-Fc than for MDTCS. The rate constant describing the disappearance of the protein in the blood (the sum of k_up_ and k_pi_ of each protein) was predicted to be 2.33 times smaller for MDTCS-Fc than for MDTCS. The kinetic modeling predicted the k_on_ value of MDTCS-Fc *in vivo* to be 6.73 × 10^−5^ nM^-1^×h^-1^ [yielding the k_off_ (=K_D_×k_on_) value of 9.69 × 10^−6^ h^-1^] (Table 3).

### Sensitivity analysis

**FIGURE 6:**
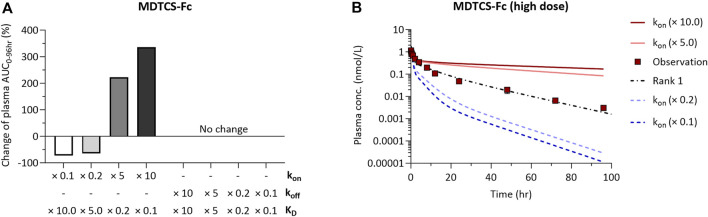
The results of local sensitivity analysis of k_on_ and k_off_ of the mechanistic kinetic model for MDTCS-Fc. The direction and magnitude of changes in AUC_0–96hr_ of parameters were similar for two doses of MDTCS-Fc. **(A)** A positive (negative) value for percentage change means an increase (decrease) in AUC_0_-_96hr_ with changes in k_on_ or k_off_. **(B)** The model-predicted plasma PK profile of MDTCS-Fc (0.0788 nmol/kg) via the 0.1, 0.2, 5.0, and 10.0-fold change of the k_on_ value. Rank 1 (black dotted line) was the accepted parameter set with the smallest SSR value. k_on_ (k_off_): The association (dissociation) rate constant between MDTCS-Fc and mouse FcRn.

We performed local sensitivity analysis to evaluate the impact of the k_on_ and k_off_ parameters for FcRn binding of MDTCS-Fc on its systemic exposure. When k_on,MDTCS-Fc_ was increased by 10-fold (with k_off,MDTCS-Fc_ unchanged, thereby the resulting K_D_ value decreased by 10-fold), the systemic exposure of MDTCS-Fc greatly (by 335.4%) increased (Figure 6A, left side). On the other hand, the systemic exposure showed no change when the same level of decrease in K_D_ (by 10-fold) was driven by a reduction of k_off,MDTCS-Fc_ (with k_on,MDTCS-Fc_ unchanged) (Figure 6A, right side). The model-predicted PK profiles using the altered k_on,MDTCS-Fc_ parameters (fold changes of 0.1–10) are shown in Figure 6B. When sensitivity analyses were performed for the other three parameters (FR_complex_, k_up,MDTCS-Fc_, k_pi,MDTCS-Fc_), the systemic exposure changed, but the magnitude of changes was much smaller than that from varying k_on,MDTCS-Fc_ (Supplementary Figure S3A). In the case of MDTCS, when k_up,MDTCS_ was decreased by 10-fold, the systemic exposure (AUC_0–24h_) increased by ∼547.6% (Supplementary Figure S3B). On the other hand, the systemic exposure (AUC_0–24h_) was not altered by varying k_pi,MDTCS_.

## Discussion

The current study evaluated the impact of Fc-fusion on the systemic PK profiles of the Fc-fusion protein and its native form using MDTCS-Fc and MDTCS. As expected, the plasma t_1/2,terminal_ of MDTCS-Fc was extended about four times compared to MDTCS (Figure 4; Table 2). Considering the sizes of both MDTCS and MDTCS-Fc being much larger than the renal glomerular filtration threshold, the prolonged circulation time of MDTCS-Fc is attributable to the intended enhancement in FcRn-mediated recycling.

The mechanistic kinetic modeling provided quantitative information regarding the FcRn-mediated recycling (Table 3). The endosomal recycling fraction of MDTCS-Fc was predicted to be 2.8-fold higher than that of MDTCS (0.8497 and 0.3061, for the low and high doses, respectively). In addition, MDTCS-Fc was predicted to undergo endocytosis at a slower rate by 7.58-fold than MDTCS (k_up_ values of 0.5296 and 4.012, respectively). It is generally known that the k_up_ was related to the pI and molecular weight of proteins (Duncan et al., 1981; Boswell et al., 2010; Hardiansyah and Ng, 2022). The theoretical pI values (based on amino acid sequences) were similar between MDTCS (pI: 6.40) and MDTCS-Fc (pI: 6.25) (prediction based on the amino acid sequences, https://web.expasy.org/compute_pi/). As such, the smaller k_up_ value of MDTCS-Fc may be attributable to its molecular weight gain over MDTCS.

The current kinetic modeling utilized the CGNM, which proves valuable for analyzing models that may not be entirely identifiable but still contain some identifiable parameters, producing simulation results with relevant information. For the kinetic models of MDTCS and MDTCS-Fc, we evaluated the identifiability of the parameters by obtaining the approximate profile likelihood (APL), as reported recently (Aoki and Sugiyama, 2023). Based on the results from the APL analysis (Supplementary Figure S4 and Supplementary Table S2), the models are not identifiable to the extent that interquartile ranges of k_pi,MDTCS_ and k_pi,MDTCS-Fc_ are unbounded within physiologically relevant limits. However, the parameters of primary interest (k_on_, k_up_, and FR) crucial to our discussions and conclusions are identifiable.

The sensitivity analyses using the kinetic model for MDTCS-Fc supported k_on,MDTCS-Fc_ as an important factor influencing the systemic exposure (Figure 6). These results may be explained by the following reasoning. The change in k_on,MDTCS-Fc_ would impact the level of the MDTCS-Fc/FcRn complex which readily transfers to either plasma or ISF (k_rc_×FR_complex_ greater than k_off,MDTCS-Fc_ by five orders of magnitude). On the other hand, the change in k_off,MDTCS-Fc_ would alter the level of the unbound MDTCS-Fc (in endosomes), which is subject to be transferred to lysosomes without being transferred to either plasma or ISF. These results may support the strategy of improving the association of Fc-fused protein therapeutics with FcRn to extend its half-life. Unfortunately, the unavailability of the MDTCS protein conjugated with wild-type or variants of Fc (of similar K_D_ values, but varying k_on_ and k_off_ values) did not allow us to experimentally verify whether improving the association of Fc-fused proteins with FcRn could extend the half-life.

It is not unexpected that the *in vitro* assay for FcRn binding may not accurately reflect *in vivo* systems. These limitations may arise in part from differences in the status of mouse FcRn protein and the binding process within the endosomes. In the *in vitro* system, the Fc component likely plays an important role in influencing FcRn binding, whereas in the more complex *in vivo* setting, other factors including the biophysical and chemical properties of molecules, may influence the binding process (Datta-Mannan and Wroblewski, 2014). As identifying *in vivo* binding kinetic parameters remains a significant challenge, the experimentally determined K_D_ values have been typically used to predict the PK profiles of antibody therapeutics (Gurbaxani et al., 2013). Our current study also utilized the K_D_ value measured *in vitro*. Following the CGNM-based optimization, the kinetic model for MDTCS-Fc yielded k_on_ and k_off_ values much lower, approximately by three orders of magnitudes, than the values obtained experimentally (Table 3; Figure 3A). Further investigations are warranted regarding the *in vitro* and *in vivo* discrepancy.

Our current kinetic models have several limitations. First, our models did not account for the potential interactions between mouse vWF and MDTCS or MDTCS-Fc. The previous study reported that the recombinant human ADAMTS13 could bind to and cleave mouse vWF (Kopić et al., 2016). As such, we cannot rule out the possibility that MDTCS or MDTCS-Fc binds to endogenous mouse vWF (target-mediated disposition). With the two dose levels tested, the PK profiles showed dose-proportionality, supporting a minimal (if any) contribution of target binding to the systemic PK profiles. Secondly, our model did not consider the pH-dependent FcRn binding as incorporated in several studies (Chen and Balthasar, 2012; Hardiansyah and Ng, 2018; Li and Balthasar, 2019; Zhang et al., 2022). Since the binding between mouse FcRn and human IgG typically displays the K_D_ values at micromolar ranges at pH 7.4 (Andersen et al., 2010; Neuber et al., 2014), we reasoned that most MDTCS-Fc would be dissociated from mouse FcRn outside endosomes. Thirdly, our model included no interference of mouse endogenous IgGs (competing MDTCS-Fc for mouse FcRn binding). But, this concern was addressed by using the value for the total FcRn concentration estimated after the binding with mouse endogenous IgGs being saturated (Ferl et al., 2005).

In summary, we constructed the mechanistic kinetic models for the Fc-fusion protein MDTCS-Fc and its native form MDTCS. In the analysis result of protein-dependent parameters, Fc-fusion reduced the rate of transfer from plasma to other space and increased the fraction of recycled. Another finding was that the association with FcRn may be an important parameter to enhance the effect of FcRn recycling. This study may enhance our understanding of the effect of Fc fusion on the PK profiles and suggest potential strategies to develop Fc-fusion therapeutics.

## Data Availability

The original contributions presented in the study are included in the article/Supplementary Material, further inquiries can be directed to the corresponding authors.
