# Supplementary material for: Kinetic modeling of the plasma pharmacokinetic profiles of ADAMTS13 fragment and its Fc-fusion counterpart in mice
Source: Front Pharmacol. 2024 Mar 25;15:1352842. doi: 10.3389/fphar.2024.1352842 (PMC10999626; doi:10.3389/fphar.2024.1352842)
Supplement: Supplementary file 1 [file DataSheet1.pdf]

# *Supplementary Material*

# Kinetic modeling of the plasma pharmacokinetic profiles of ADAMTS13 fragment and its Fc-fusion counterpart in mice


**Heechun Kwak**

*** Correspondence:**

Hyun-Ja Nam, hjnam098@gccorp.com

Wooin Lee, wooin.lee@snu.ac.kr


## Supplementary method

### Model equations

$C_Z$ and $V_Z$ described the concentration and volume of each compartment. Z refer to 1 = plasma, 2 = endosome, 3 = ISF space, 4 = lymph. $C2_{complex}$ and $C2_{MDTCS\text{-}Fc}$ are the concentrations of bound and unbound MDTCS-Fc in endosomes. The following set of equations calculated the concentration of MDTCS or MDTCS-Fc:

[MDTCS]

$$dC1_{MDTCS}/dt = 1/V1 \times \left[FR_{MDTCS} \times k_{rc} \times V2 \times C2_{MDTCS} + L \times C4_{MDTCS} - (k_{up,MDTCS} + k_{pi,MDTCS}) \times V1 \times C1_{MDTCS}\right]$$

$$dC2_{MDTCS}/dt = 1/V2 \times \left[k_{up,MDTCS} \times V1 \times C1_{\,MDTCS} + k_{up,MDTCS} \times V3 \times C3_{\,MDTCS} - FR_{MDTCS} \times k_{rc} \times V2 \times C2_{MDTCS} - (1 - FR_{MDTCS}) \times k_{rc} \times V2 \times C2_{MDTCS} - k_e \times V2 \times C2_{MDTCS}\right]$$

$$dC3_{MDTCS}/dt = 1/V3 \times \left[k_{up,MDTCS} \times V1 \times C1_{\,MDTCS} + (1 - FR_{MDTCS}) \times k_{rc} \times V2 \times C2_{MDTCS} - k_{up,MDTCS} \times V3 \times C3_{\,MDTCS} - L \times C3_{\,MDTCS}\right]$$

$$dC4_{MDTCS}/dt = 1/V4 \times (L \times C3_{MDTCS} - L \times C4_{MDTCS})$$

[MDTCS-Fc]

$$dC1_{MDTCS-Fc}/dt = 1/V1 \times \left[FR_{complex} \times k_{rc} \times V2 \times C2_{complex} + L \times C4_{MDTCS-Fc} - \left(k_{up,MDTCS-Fc} + k_{pi,MDTCS-Fc}\right) \times V1 \times C1_{MDTCS-Fc}\right]$$

$$dC2_{complex}/dt = 1/V2 \times \left[k_{on,MDTCS-Fc} \times V2 \times C2_{MDTCS-Fc} \times FcRn_{free} - FR_{complex} \times k_{rc} \times V2 \times C2_{complex} - \left(1 - FR_{complex}\right) \times k_{rc} \times V2 \times C2_{complex} - \left(K_D \times k_{on,MDTCS-Fc}\right) \times V2 \times C2_{complex}\right]$$

$$dC2_{MDTCS-Fc}/dt = 1/V2 \times \left[k_{up,MDTCS-Fc} \times V1 \times C1_{MDTCS-Fc} + k_{up,MDTCS-Fc} \times V3 \times C3_{MDTCS-Fc} + \left(K_D \times k_{on,MDTCS-Fc}\right) \times V2 \times C2_{complex} - k_{on,MDTCS-Fc} \times V2 \times C2_{MDTCS-Fc} \times FcRn_{free} - k_e \times V2 \times C2_{MDTCS-Fc}\right]$$

$$dC3_{MDTCS-Fc}/dt = 1/V3 \times \left[(1 - FR_{complex}) \times k_{rc} \times V2 \times C2_{complex} + k_{pi,MDTCS-Fc} \times V1 \times C1_{MDTCS-Fc} - k_{up,MDTCS-Fc} \times V3 \times C3_{MDTCS-Fc} - L \times C3_{MDTCS-Fc}\right]$$

$$dC4_{MDTCS-Fc}/dt = 1/V4 \times (L \times C3_{MDTCS-Fc} - L \times C4_{MDTCS-Fc})$$

$$dFcRn_{free}/dt = 1/V2 \times \left[\left(K_D \times k_{on,MDTCS-Fc}\right) \times V2 \times C2_{complex} - k_{on,MDTCS-Fc} \times V2 \times C2_{MDTCS-Fc} \times FcRn_{free}\right]$$

**Sensitivity analysis**

To identify sensitive parameters that affect the model, a local sensitivity analysis was performed on the final model and parameters. All the protein-dependent optimized parameters were subjects for the analysis, $k_{up,MDTCS}$, $k_{pi,MDTCS}$, and $FR_{MDTCS}$ for MDTCS, and $k_{up,MDTCS-Fc}$, $k_{pi,MDTCS-Fc}$, $FR_{complex}$, $k_{on,MDTCS-Fc}$ and $k_{off,MDTCS-Fc}$ were for MDTCS-Fc. The area under the curve to the last time point ($AUC_{0-24hr}$ for MDTCS, $AUC_{0-96hr}$ for MDTCS-Fc) of the plasma concentration vs. time curve was selected as the relevant model output to represent drug exposure. We estimated the sensitivity of each parameter with the percentage change in $AUC_{last}$ by changing the drug-related parameters of the kinetic model by 0.1-, 0.2-, 5-, and 10-fold. (Shah and Betts 2012). However, since FR cannot be greater than ‘1’, it was set to ‘1’ for the local sensitivity analysis instead of 5- or 10-fold changes.

$$\%change = \frac{AUC_{chg} - AUC_{sim}}{AUC_{sim}} \times 100$$

$AUC_{sim}$ is the $AUC_{last}$ obtained with the Rank1 parameter set and $AUC_{chg}$ is the $AUC_{last}$ obtained with altered parameter values. The analysis was performed on all the datasets at the same dose. We simulated changed parameter values with Berkeley Madonna version 10.4.4 (University of California, Berkeley, U.S.). Exported data from Berkeley Madonna was plotted using GraphPad Prism version 10.0.2 (GraphPad Software, California, U.S.).

# 1 Supplementary Figures and Tables

## 1.1 Supplementary Figures

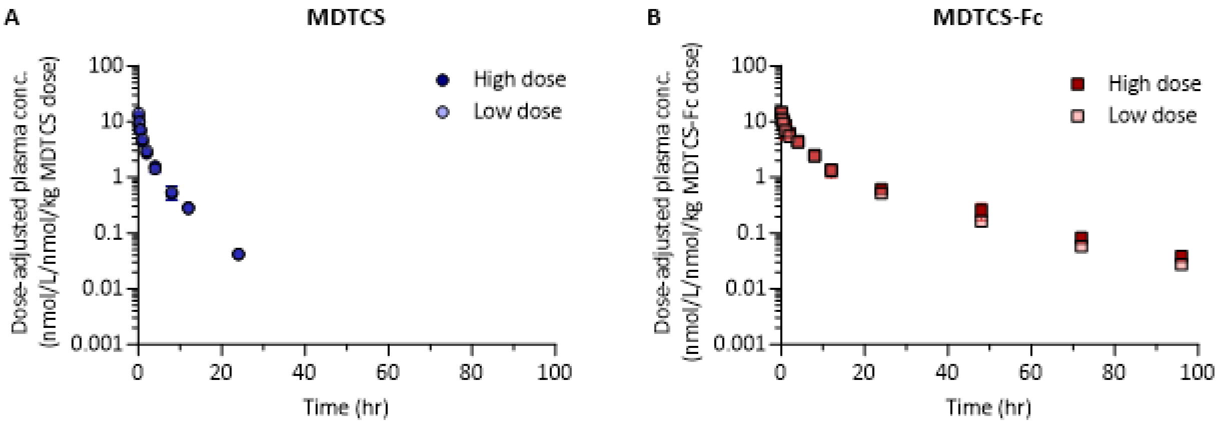


**Supplementary Figure S1.** The dose-adjusted plasma pharmacokinetic profiles of (A) MDTCS after a single i.v. dosing of 0.39 or 0.78 nmol/kg and (B) MDTCS-Fc after a single i.v. dosing of 0.0394 or 0.0788 nmol/kg in mice. Each data point represents the mean concentration with the corresponding standard deviation measured using the plasma samples collected via heart puncture (n=3-4 mice per time point).

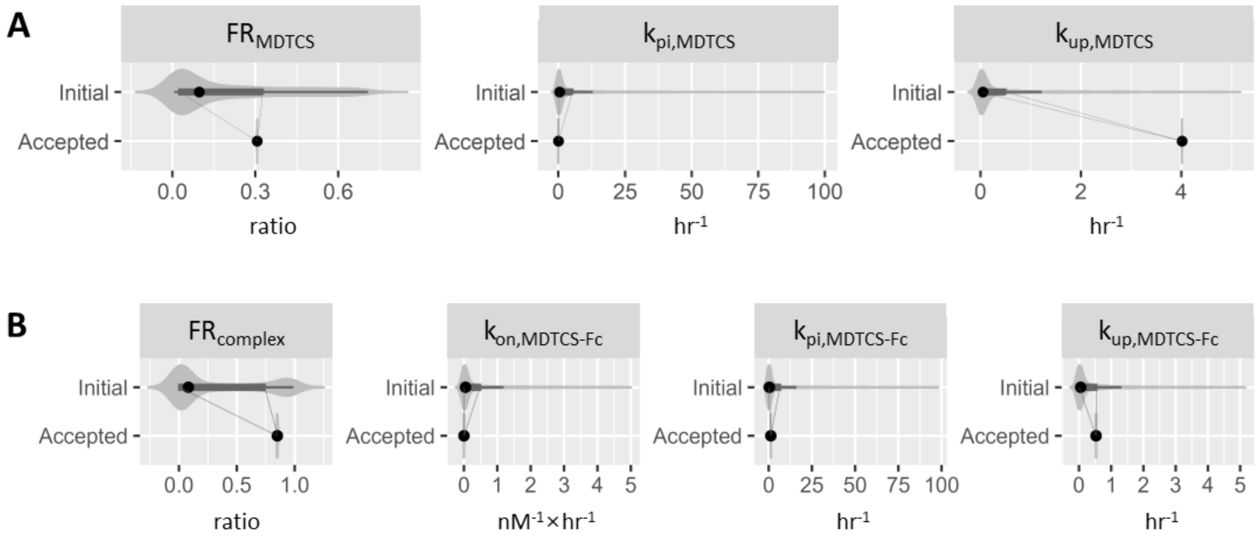


**Supplementary Figure S2.** The distribution of the initial and accepted parameter sets from Cluster Gauss-Newton Method (CGNM) runs of (A) MDTCS and (B) MDTCS-Fc. Median values are represented by closed circles, with quartile values indicated by solid lines. $k_{up}$: endothelial cell pinocytosis rate constant, $k_{pi}$: the rate constant from plasma to interstitial fluid, $k_{on}$ ($k_{off}$): The association (dissociation) rate constant between MDTCS-Fc and mouse FcRn, FR: fraction of recycled therapeutics to plasma.

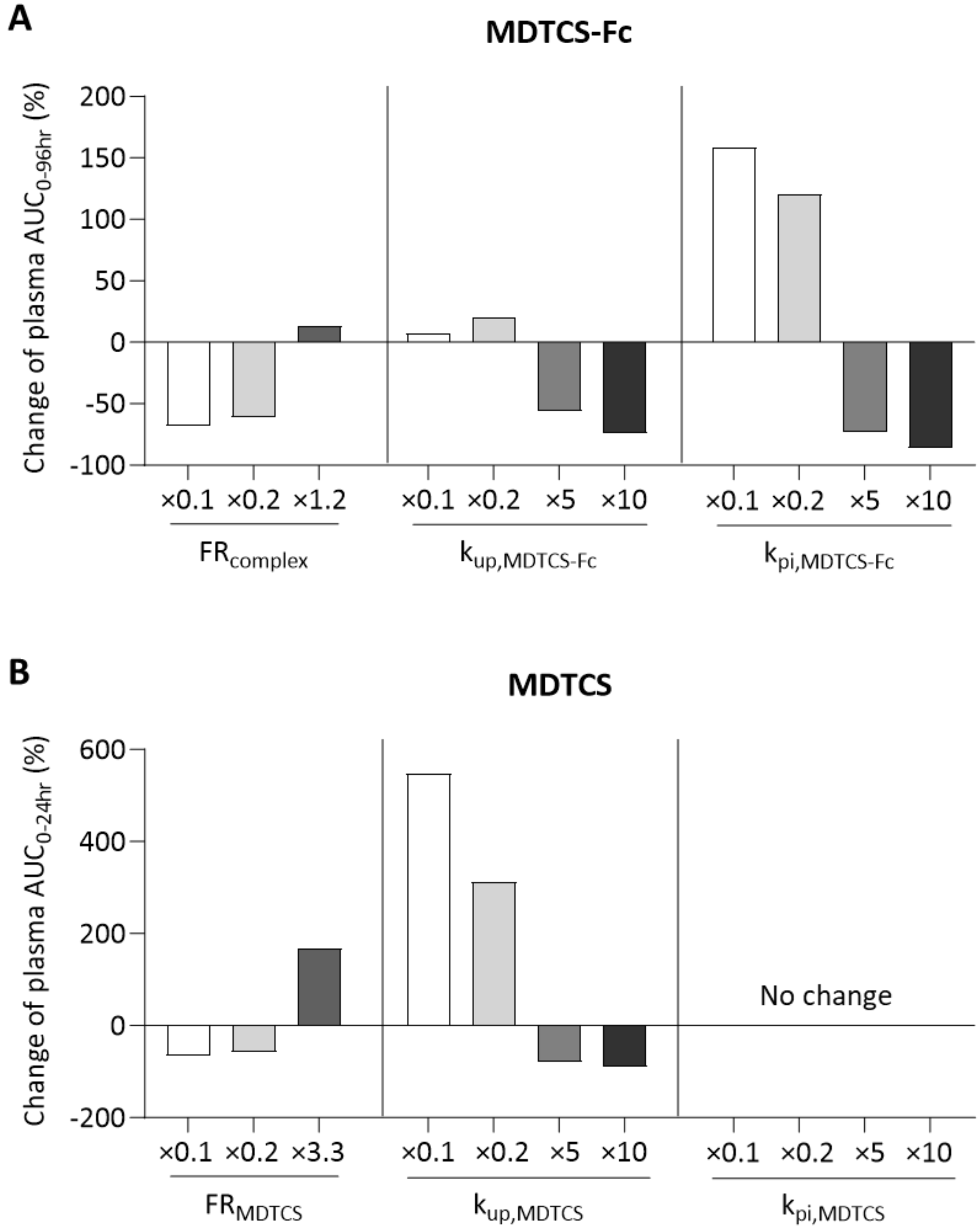


**Supplementary Figure S3.** The result of local sensitivity analysis of drug-related parameters of the kinetic model for (A) MDTCS-Fc and (B) MDTCS. The direction and strength of changes in $AUC_{chg}$ of parameters were very similar for two doses of each drug. A positive (negative) value for percentage change means an increase (decrease) in $AUC_{last}$ with changes in parameter values. $k_{up}$: endothelial cell endocytosis rate constant, $k_{pi}$: the rate constant from plasma to interstitial fluid, $k_{on}$ ($k_{off}$): The association (dissociation) rate constant between MDTCS-Fc and FcRn, FR: fraction of recycled therapeutics to plasma.

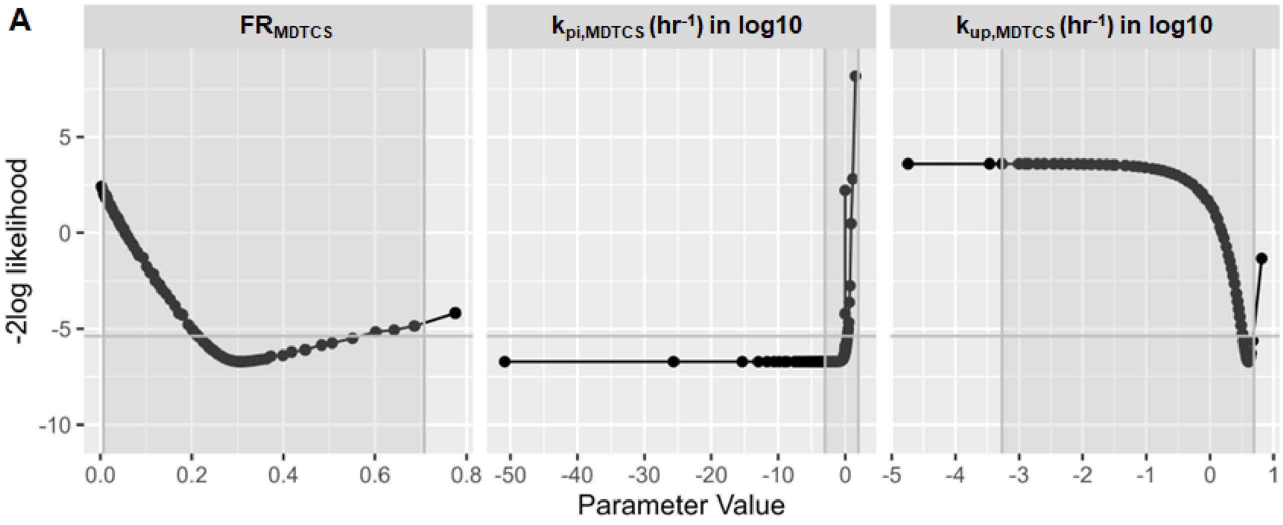


Horizontal grey line is the threshold for confidence intervals corresponding to a confidence level of 75%.

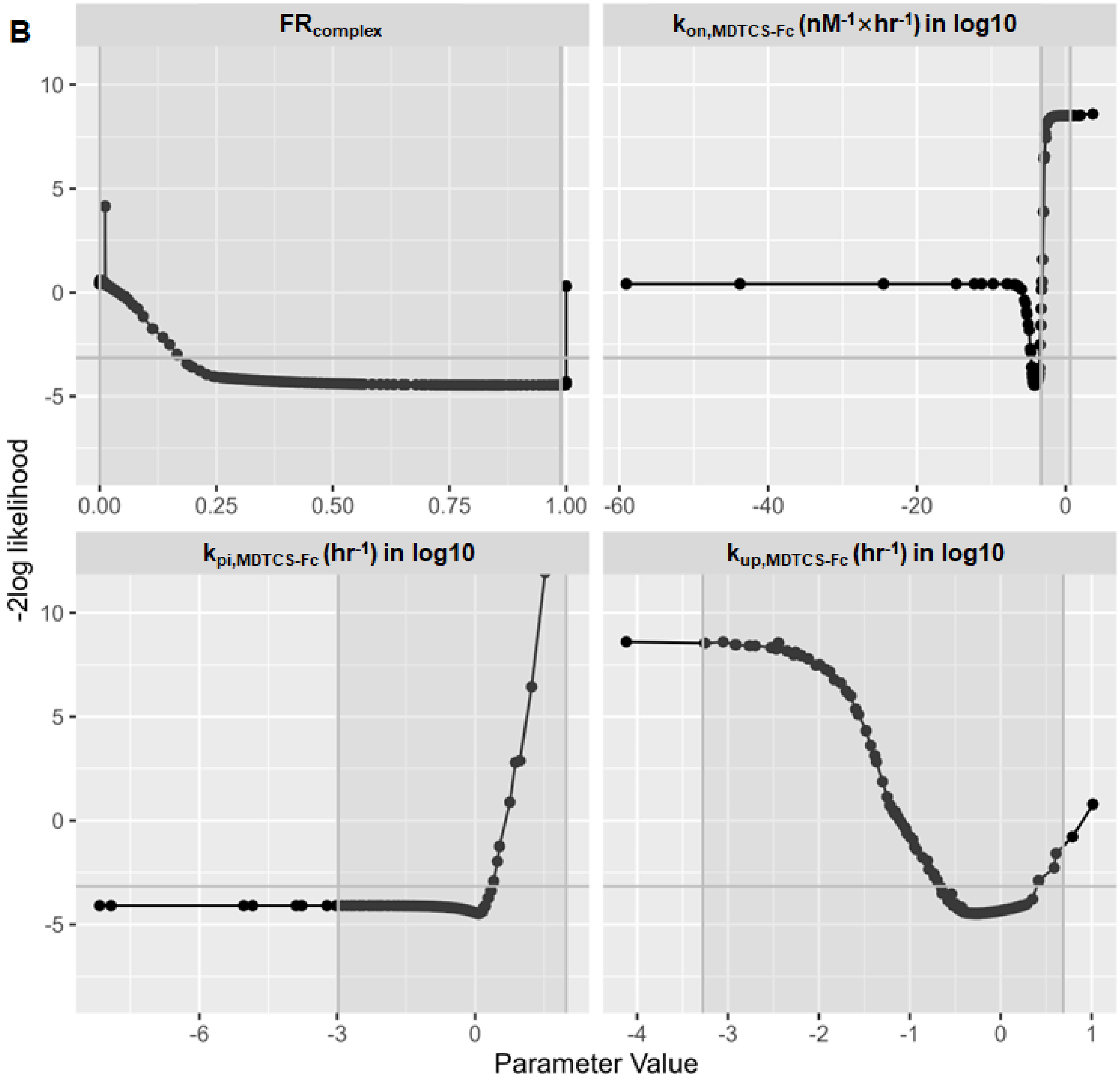


Horizontal grey line is the threshold for confidence intervals corresponding to a confidence level of 75%.

**Supplementary Figure S4.** The plot of approximate profile likelihoods of the kinetic model for (A) MDTCS and (B) MDTCS-Fc. The black solid lines and dots represent the approximate profile likelihood calculated via Cluster Gauss-Newton method. Vertical grey lines are the initial ranges of each parameter. $k_{up}$: endothelial cell endocytosis rate constant, $k_{pi}$: the rate constant from plasma to interstitial fluid, $k_{on}$ ($k_{off}$): The association (dissociation) rate constant between MDTCS-Fc and FcRn, FR: fraction of recycled therapeutics to plasma

## 1.2 Supplementary Tables

**Supplementary Table S1.** Accuracy and precision of sandwich ELISA for MDTCS and MDTCS-Fc in mouse plasma. The calculated data represents mean ± standard deviation or mean with raw data (numbers in the parentheses).

| **MDTCS** | Concentration (pM) | | Accuracy (%) | CV (%) |
|---|---|---|---|---|
| | Theoretical | Calculated | | |
| Intra-assay | 2.5 | 2.75 ± 0.11 | 110.0 | 4.0 |
| n=4 | 7.5 | 8.12 ± 0.11 | 108.3 | 1.4 |
| | 25.0 | 23.60 ± 0.29 | 94.4 | 1.2 |
| | 50.0 | 52.72 ± 0.29 | 105.4 | 0.6 |
| Inter-assay | 2.5 | 2.37 (2.36, 2.38) | 94.8 | 0.4 |
| n=2 | 7.5 | 7.13 (7.01, 7.25) | 95.0 | 2.4 |
| | 25.0 | 23.17 (22.86, 23.47) | 92.7 | 1.9 |
| | 50.0 | 47.84 (46.93, 48.75) | 95.7 | 2.7 |

| **MDTCS-Fc** | Concentration (pM) | | Accuracy (%) | CV (%) |
|---|---|---|---|---|
| | Theoretical | Calculated | | |
| Intra-assay | 0.227 | 0.234 (0.232, 0.236) | 103.0 | 1.1 |
| n=2 | 0.682 | 0.698 (0.691, 0.705) | 102.3 | 1.2 |
| | 2.273 | 2.418 (2.400, 2.436) | 106.4 | 1.0 |
| | 4.545 | 4.677 (4.636, 4.718) | 102.9 | 1.2 |
| Inter-assay | 0.227 | 0.234 (0.234, 0.234) | 103.0 | 0.0 |
| n=2 | 0.682 | 0.699 (0.698, 0.700) | 102.5 | 0.2 |
| | 2.273 | 2.422 (2.418, 2.425) | 106.6 | 0.2 |
| | 4.545 | 4.783 (4.677, 4.889) | 105.2 | 3.1 |

**Supplementary Table S2.** The profile likelihood confidence interval of the kinetic model for MDTCS and MDTCS-Fc. The interquartile ranges are represented by square brackets, which show the upper or lower limits based on the profile likelihood. If the upper or lower limits of the interquartile ranges extend beyond the domain of the profile likelihood, then they are indicated by parentheses (Aoki and Sugiyama 2023). NA: not applicable

| **Proteins administered** | **Parameter** | **Best-fit [25 percentile, 75 percentile]** | **Identifiability** |
|---|---|---|---|
| MDTCS | $FR_{MDTCS}$ | 0.3061 [0.212, 0.576] | Identifiable |
| | $k_{up,MDTCS}$ ($hr^{-1}$) | 4.012 [3.308, 5.621] | Identifiable |
| | $k_{pi,MDTCS}$ ($hr^{-1}$) | NA (< $1.382\times10^{-51}$, 2.065] | Not identifiable |
| MDTCS-Fc | $FR_{complex}$ | 0.8497 [0.177, 1.000] | Identifiable |
| | $k_{up,MDTCS\text{-}Fc}$ ($hr^{-1}$) | 0.5296 [0.211, 2.438] | Identifiable |
| | $k_{pi,MDTCS\text{-}Fc}$ ($hr^{-1}$) | 1.191 (< $6.794\times10^{-9}$, 2.363] | Not identifiable |
| | $k_{on,MDTCS\text{-}Fc}$ ($nM^{-1}\times hr^{-1}$) | $6.729\times10^{-5}$ [$2.396\times10^{-5}$, $3.220\times10^{-4}$] | Identifiable |

**References**

Aoki, Y. and Y. Sugiyama (2023). "Cluster Gauss-Newton method for a quick approximation of profile likelihood: With application to physiologically-based pharmacokinetic models." CPT Pharmacometrics Syst Pharmacol.

Shah, D. K. and A. M. Betts (2012). "Towards a platform PBPK model to characterize the plasma and tissue disposition of monoclonal antibodies in preclinical species and human." J Pharmacokinet Pharmacodyn **39**(1): 67-86.
